# Giving short‐term prophylactic antibiotics in patients undergoing open and laparoscopic hepatic resection

**DOI:** 10.1002/ags3.12267

**Published:** 2019-05-30

**Authors:** Hiroji Shinkawa, Shogo Tanaka, Shigekazu Takemura, Ryosuke Amano, Kenjiro Kimura, Takayoshi Nishioka, Tokuji Ito, Toru Miyazaki, Atsushi Ishihara, Shoji Kubo

**Affiliations:** ^1^ Department of Hepato‐Biliary‐Pancreatic Surgery Osaka City University Graduate School of Medicine Osaka Japan

**Keywords:** antibiotics, antimicrobial, incidence, propensity score, surgical site infection

## Abstract

**Aim:**

The 2016 guidelines of the Japan Society for Surgical Infection and the Japan Society of Chemotherapy advocate giving prophylactic antibiotics 1 hour before surgery and until 24 hours after surgery in patients undergoing elective hepatic resection. However, the efficacy of short‐term antimicrobial prophylaxis has not been evaluated according to surgical approach. We evaluated the efficacy of giving prophylactic antibiotics in patients undergoing open or laparoscopic hepatic resection.

**Methods:**

The study comprised 218 and 185 patients undergoing open and pure laparoscopic hepatic resection, respectively. Incidence rates of postoperative infectious complications were compared between patients who received flomoxef sodium as the prophylactic antibiotic before and until 24 hours after surgery (short‐term group) and those who received flomoxef sodium until 72 hours after surgery (long‐term group) among patients undergoing open or laparoscopic hepatic resection. Propensity score matching analysis was carried out to adjust for confounding factors between the short‐ and long‐term groups.

**Results:**

There was no significant difference in the postoperative infectious complication incidence between the short‐ and long‐term groups among patients undergoing open (18.9% vs 12.2%; *P *=* *0.36) or laparoscopic (3.3% vs 1.7%; *P *>* *0.99) hepatic resection after propensity score matching. Incidence rate of surgical site infections was comparable between the short‐ and long‐term groups among patients undergoing open (13.5% vs 10.8%; *P *=* *0.80) or laparoscopic (3.3% vs 1.7%; *P *>* *0.99) hepatic resection.

**Conclusion:**

Giving short‐term prophylactic antibiotics might be sufficient in preventing postoperative infectious complications in patients undergoing open and laparoscopic hepatic resection.

## 1 INTRODUCTION

Improvements in surgical techniques and perioperative management of patients undergoing hepatic resection without biliary tract reconstruction have reduced surgical mortality rates from 10% to 2%‐4% in the last two decades.^1^, ^2^ Despite the improvement in operative mortality, postoperative complications occur in 20%‐30% of patients after hepatic resection.^3^, ^4^ Infectious complications including surgical site infections (SSI) and remote site infections (RSI) are common causes of major morbidity and occur in 5%‐20% of patients with hepatic resection.^5^, ^6^, ^7^ In 1999, the Centers for Disease Control and Prevention (CDC) provided extensive recommendations for SSI control.^8^ Specifically, antimicrobial prophylaxis was recommended to prevent postoperative SSI. In subsequent guidelines, giving prophylactic antibiotics was recommended to be started within 1 hour before making the skin incision,^9^, ^10^, ^11^, ^12^ with redosing during surgery^9^, ^10^, ^11^ and discontinuation within 24 hours after surgery.^9^, ^10^, ^11^

Hepatic resection is classified as a clean‐contaminated surgical procedure because the bile duct is dissected. Appropriate timing of giving prophylactic antibiotics has been investigated in several studies.^6^, ^13^, ^14^ A recent nationwide randomized controlled study by the Japan Society for Surgical Infection (JSSI) showed that the efficacy of prophylactic antibiotic use for SSI prevention by dosing before and until 24 hours after hepatic resection for hepatocellular carcinoma was not inferior to dosing before and 72 hours after surgery.^15^ JSSI and the Japan Society of Chemotherapy (JSC) guidelines recommend starting antibiotics before surgery, with redosing every 3 hours during surgery and continuation until 24 hours after hepatic resection.^16^

Laparoscopic hepatic resection (LHR) is a commonly accepted treatment option for both benign and malignant liver diseases. Numerous reports have indicated the advantages of LHR such as reduced surgical stress, intraoperative blood loss, and postoperative morbidity including SSI in comparison with open hepatic resection (OHR).^17^, ^18^, ^19^ Therefore, the efficacy of giving prophylactic antibiotics should be validated according to the surgical approach including laparoscopic and open surgical approaches. However, no study has investigated the efficacy of giving short‐term prophylactic antibiotics in patients undergoing LHR. In the present study, we validated the efficacy of giving short‐term prophylactic antibiotics in patients undergoing OHR by comparing the incidence of postoperative infectious complications (PIC) including SSI between patients who received prophylactic antibiotics before and until 24 hours after surgery and those who received the antibiotics before and until 72 hours after surgery. Additionally, we evaluated the effect of giving short‐term prophylactic antibiotics in patients undergoing LHR.

## 2 MATERIALS AND METHODS

### 2.1 Patients

This retrospective study included 433 patients who underwent hepatic resection for liver tumors with no biliary reconstruction or resection of other organs at the Osaka City University Hospital from April 2012 to December 2017. To clarify the true postoperative outcome in patients with LHR, we excluded patients who underwent laparoscopy‐assisted hepatic resection (n = 24) and hand‐assisted laparoscopic resection (n = 6) in the present study. Of the remaining 403 patients, 218 patients underwent OHR, and 185 patients underwent pure laparoscopic resection. Primary diagnoses included hepatocellular carcinoma (n = 313), intrahepatic cholangiocarcinoma (n = 31), mixed‐type liver cancer (n = 8), biliary cystadenocarcinoma (n = 2), metastatic liver tumor (n = 25), malignant lymphoma (n = 2), angiosarcoma (n = 1), leiomyosarcoma (n = 1), angiomyolipoma (n = 1), hemangioma (n = 10), focal nodular hyperplasia (n = 3), inflammatory pseudotumor (n = 3), and other benign disease (n = 3). All clinical data including history of the presenting illness, physical examination, laboratory test results, and pathological findings were obtained from the medical records. The study was conducted following the guidelines of the Ethics Committee of Osaka City University (No. 3815) and the Declaration of Helsinki, after approval by the Ethics Committee. Informed consent was obtained from all patients.

### 2.2 Protocol for antibiotic prophylaxis

As a prophylactic antibiotic, 1.0 g flomoxef sodium (FMOX) was given 1 hour before the skin incision. The agent was redosed at 3‐h intervals from the time of preoperative dosing and given at 3 hours after the completion of surgery. From January 2012 to March 2014, FMOX was given every 12 hours for 3 days after surgery (long‐term group), whereas after April 2014, FMOX was given before and within 24 hours after surgery (short‐term group).

### 2.3 Operative procedures

For OHR, iodophor solutions were used for preoperative skin preparation at the incision site. The incision site was covered with a plastic adhesive drape impregnated with the iodophor (Ioban 2; 3M Health Care, Tokyo, Japan).^20^ Hepatic parenchymal transection was done using an ultrasonic dissector (CUSA Excel; Integra LifeSciences, Plainsboro Township, NJ, USA). Intrahepatic vessels were coagulated using the VIO soft‐coagulation system (VIO 300D; ERBE Elektromedizin, Tübingen, Germany) and ligated with absorbable threads. Hepatic inflow was occluded before hepatic resection by the Pringle maneuver (15‐minutes occlusion and 5‐minutes reperfusion). A bile leakage test using balloon catheter was carried out in patients who underwent cholecystectomy. A 6‐Fr transcystic duct tube (C‐tube, Sumius; Sumitomo Bakelite Co., Tokyo, Japan) was placed if postoperative bile leakage was expected.^21^ One or two closed abdominal drains (BLAKE silicon drains and J‐VAC reservoirs; Johnson & Johnson Medical, New Brunswick, NJ, USA) were generally inserted near the liver transection plane or into the foramen of Winslow. Non‐placement of abdominal drain was permitted at surgeon's discretion. After closure of the abdominal fascia with interrupted sutures using 0 PDS II threads (Johnson & Johnson Medical), the surgical incision was irrigated with 500 mL saline. The skin was closed using an interrupted subcuticular suture using 4‐0 PDS II threads. For LHR, three to six trocars were inserted using an open technique. Port location was based on the operation type. Hepatic parenchymal transection was done using a laparoscopic ultrasonic surgical aspirator and bipolar or monopolar forceps with the VIO soft‐coagulation system. The Pringle maneuver was carried out using a temporary endoscopic clip. The resected specimen was placed into a plastic bag and externalized through the slightly extended port site. One or two closed abdominal drains were generally inserted near the liver transection plane or into the foramen of Winslow. Non‐placement of abdominal drain was permitted at surgeon's discretion. Type of hepatic resection was classified according to The Brisbane 2000 Terminology of Liver Anatomy and Resections.^22^

### 2.4 Postoperative management

Patients were allowed to drink on postoperative day (POD) 1 and take food orally on POD 2. Urinary catheter was routinely removed on POD 3. The central line was usually removed on POD 3 or 4 if the central catheter was inserted preoperatively. Abdominal drains were removed on POD 2 or 3 after confirming hemostasis and the absence of bile leakage. Postoperative bile leakage was defined according to the International Study Group of Liver Surgery.^23^ If the drain fluid was bile‐stained or contaminated, the drain was retained until the bile leakage or abdominal infection resolved. The C‐tube, if inserted, was removed on POD 7 or 8 after confirming the absence of bile leakage. All medical staff including physicians and nurses complied strictly with the standard precautions provided by the CDC to prevent the transmission of infectious agents at the hospital.^24^

### 2.5 Definition of infections

Infectious complications were defined as combined SSI and RSI. SSI were defined as infections occurring within 30 days after surgery at any incision or abdominal space according to the CDC guidelines.^8^ RSI were defined based on the presence of fever and leukocytosis with bacteria in urine, sputum, catheter tip, blood, or bile juice or the physician's judgment regardless of microbiological evidence.

For diagnosis of *Clostridioides difficile* infection, glutamate dehydrogenase (GDH) and toxin A/B assays were used.^25^ *Clostridioides difficile* colitis was diagnosed based on the presence of diarrhea and the detection of the toxin and GDH antigen produced by *C. difficile* in the stool of the patient.

### 2.6 Propensity score matching

We carried out propensity score matching (PSM) to adjust for potential confounders between the short‐ and long‐term groups among patients undergoing OHR and LHR.^26^ Variables entered in the propensity model were age, gender, presence of diabetes mellitus, body mass index, American Society of Anesthesiologists (ASA) class, alanine aminotransferase (ALT) level, platelet count, Child‐Pugh grade, and major hepatectomy (excision of ≥2 sections). We calculated propensity scores using a logistic regression model including these variables. The C statistic was calculated to evaluate the goodness of fit. We carried out one‐to‐one PSM without replacement using a caliper width of 0.25 standard deviation of the logit of propensity score. We compared pathological and perioperative outcomes between the short‐ and long‐term groups including the presence of liver cirrhosis, operative time (<8 or ≥8 hours), blood loss (<500 or ≥500 mL), postoperative complications (≥Clavien‐Dindo grade II), bile leakage, and infectious complications, including SSI and RSI.

### 2.7 Statistical analysis

Categorical variables were compared using the chi‐squared or Fisher's exact probability tests. All statistical analyses were carried out using SPSS statistical software version 22.0 (SPSS). The threshold for significance was set at a *P* value of <0.05.

## 3 RESULTS

Table 1 shows the background characteristics and surgical outcomes of the patients undergoing OHR and LHR. The proportion of patients with a platelet count <10 × 10^4^/μL was higher in the LHR group; however, the proportion of patients with major hepatectomy was higher in the OHR group. Among the surgical outcomes, the proportions of patients with longer operative times (≥8 hours), more intraoperative blood loss (≥500 mL), worse postoperative complications (≥Clavien‐Dindo grade II), infectious complications, and SSI were higher among those undergoing OHR. Of the 403 patients, SSI were observed in 32 patients (7.9%), including nine patients with incisional SSI and 27 patients with organ/space SSI. Table 2 shows the microorganisms isolated from the surgical sites. Methicillin‐resistant *Staphylococcus aureus* (MRSA) was isolated in four patients (1.0%), including two patients with incisional SSI and two patients with organ/space SSI.

**Table 1:** Comparison of background characteristics and surgical outcomes between patients with open hepatic resection (OHR) and laparoscopic hepatic resection (LHR)

	OHR (n = 218) (%)	LHR (n = 185) (%)	*P*
Age >65 y	160 (73.4)	127 (68.6)	0.32
Gender, male	157 (72.0)	131 (70.8)	0.83
Diabetes	71 (32.6)	64 (34.6)	0.67
BMI >25 kg/m^2^	63 (28.9)	62 (33.5)	0.33
ASA class ≥3	33 (15.1)	32 (17.3)	0.59
ALT >30 U/L	87 (39.9)	87 (47.0)	0.16
Platelet count <10 × 10^4^/μL	20 (9.2)	41 (22.2)	<0.001
Child‐Pugh grade B	8 (3.7)	8 (4.3)	0.80
Major hepatectomy	83 (38.1)	8 (4.3)	<0.001
Operation type			<0.001
Limited resection	69 (31.7)	154 (83.2)	
Segmentectomy	22 (10.1)	4 (2.2)	
Left lateral sectionectomy	7 (3.2)	17 (9.2)	
Left medial sectionectomy	9 (4.1)	0 (0)	
Right anterior sectionectomy	15 (6.9)	1 (0.5)	
Right posterior sectionectomy	13 (6.0)	2 (1.1)	
Hemihepatectomy	74 (33.9)	7 (3.8)	
Trisectionectomy	3 (1.4)	0 (0)	
Central bisectionectomy	6 (2.8)	0 (0)	
Operative time ≥8 h	38 (17.4)	9 (4.9)	<0.001
Intraoperative blood loss ≥500 mL	108 (49.5)	9 (4.9)	<0.001
Abdominal drainage	204 (93.6)	159 (85.9)	0.012
Postoperative complications (≥Clavien‐Dindo grade II)	84 (38.5)	16 (8.6)	<0.001
Infectious complications	36 (16.5)	3 (1.6)	<0.001
SSI	29 (13.3)	3 (1.6)	<0.001
Incisional SSI	9 (4.1)	0 (0)	0.062
Organ/space SSI	24 (11.0)	3 (1.6)	<0.001

Abbreviations: ALT, alanine aminotransferase; ASA, American Society of Anesthesiologists; BMI, body mass index; SSI, surgical site infection.

**Table 2:** Microorganisms isolated from the surgical sites

Infection site	All SSI (n = 32)	
	Micro‐organism	No.
Incision (n = 9)	MRSA	2
	MRCNS	1
	CNS	2
	*Staphylococcus aureus*	1
	*Enterococcus species*	1
	*Enterobacter cloacae*	2
Organ/space (n = 27)	*Enterococcus faecalis*	6
	*Enterobacter cloacae*	4
	MRCNS	3
	*Staphylococcus aureus*	4
	MRSA	2
	*Klebsiella pneumoniae*	1
	*Candida glabrata*	1
	*Streptococcus salivarius*	1
	*Pseudomonas aeruginosa*	2
	*Bacteroides fragilis*	1
	*Enterococcus* species	1
	*Corynebacterium* species	1

Abbreviations: CNS, coagulase‐negative staphylococci; MRCNS, methicillin‐resistant coagulase‐negative staphylococci; MRSA, methicillin‐resistant *Staphylococcus aureus*; SSI, surgical site infection.

Table 3 shows the background characteristics of the patients undergoing OHR in the short‐ and long‐term groups of the unmatched and the matched cohorts. In the unmatched cohort, abdominal drainage tube was more frequently inserted in the short‐term group, whereas the proportion of males tended to be higher in the long‐term group. After PSM, the study group of 148 patients was well matched. Proportion of abdominal drainage was comparable between the two groups. Pathological diagnosis of liver cirrhosis was confirmed in nine patients (12.2%) in the short‐term group and 13 patients (17.6%) in the long‐term group (*P *=* *0.49).

**Table 3:** Background characteristics of patients undergoing open hepatic resection

	Unmatched cohort			Matched cohort		
	Short‐term group (n = 143) (%)	Long‐term group (n = 75) (%)	*P*	Short‐term group (n = 74) (%)	Long‐term group (n = 74) (%)	*P*
Age >65 y	109 (76.2)	51 (68.0)	0.20	55 (74.3)	50 (67.6)	0.47
Gender, male	109 (76.2)	48 (64.0)	0.059	48 (64.9)	48 (64.9)	>0.99
Diabetes	44 (30.8)	27 (36.0)	0.45	26 (35.1)	27 (36.5)	>0.99
BMI >25 kg/m^2^	40 (28.0)	23 (30.7)	0.75	22 (29.7)	22 (29.7)	>0.99
ASA class ≥3	25 (17.5)	8 (10.7)	0.23	6 (8.1)	8 (10.8)	0.78
Platelet count <10 × 10^4^/μL	11 (7.7)	9 (12.0)	0.20	6 (8.1)	8 10.8)	0.78
ALT >30 U/L	54 (37.8)	33 (44.0)	0.39	36 (48.6)	32 (43.2)	0.62
Child‐Pugh grade B	6 (4.2)	2 (2.7)	0.72	1 (1.4)	2 (2.7)	>0.99
Major hepatectomy	58 (40.6)	25 (33.3)	0.31	28 (37.8)	25 (33.8)	0.73
Operation type			0.30			0.54
Limited resection	41 (28.7)	28 (37.3)		25 (33.8)	27 (36.5)	
Segmentectomy	11 (7.7)	11 (14.7)		6 (8.1)	11 (14.9)	
Left lateral sectionectomy	6 (4.2)	1 (1.3)		3 (4.1)	1 (1.4)	
Left medial sectionectomy	6 (4.2)	3 (4.0)		4 (5.4)	3 (4.1)	
Right anterior sectionectomy	10 (7.0)	5 (6.7)		3 (4.1)	5 (6.8)	
Right posterior sectionectomy	11 (7.7)	2 (2.7)		5 (6.8)	2 (2.7)	
Hemihepatectomy	52 (36.4)	22 (29.3)		25 (33.8)	22 (29.7)	
Trisectionectomy	1 (0.7)	2 (2.7)		0 (0)	2 (2.7)	
Central bisectionectomy	5 (3.5)	1 (1.3)		3 (4.1)	1 (1.4)	
Abdominal drainage	138 (96.5)	66 (88.0)	0.02	70 (94.6)	66 (89.2)	0.37

Abbreviations: ALT, alanine aminotransferase; ASA, American Society of Anesthesiologists; BMI, body mass index.

Table 4 shows the perioperative outcomes in patients undergoing OHR in the short‐ and long‐term groups of the unmatched and the matched cohorts. Proportion of greater intraoperative blood loss (≥500 mL) was higher in the short‐term group of the unmatched cohort. However, the proportion of patients with greater intraoperative blood loss (≥500 mL) was comparable between the short‐ and long‐term groups (50.0% vs 40.5%, *P *=* *0.32) in the matched cohort, similar to those observed for longer operative times (≥8 hours; 16.2% vs 17.6%, *P *>* *0.99) and worse postoperative complications (≥Clavien‐Dindo grade II; 36.5% vs 33.8%, *P *=* *0.86). There was no significant difference in the incidence rates of infectious complications (18.9% vs 12.2%, *P *=* *0.36) or SSI (13.5% vs 10.8%, *P *=* *0.80). *Clostridioides* colitis developed in only two patients (1.4%) in the short‐term group. These patients were given proton pump inhibitors starting from POD 2, presented with diarrhea at POD 7, and were positive by the *Clostridioides* toxin and GDH assays.

**Table 4:** Perioperative outcomes in patients undergoing open hepatic resection

	Unmatched cohort			Matched cohort		
	Short‐term group (n = 143) (%)	Long‐term group (n = 75) (%)	*P*	Short‐term group (n = 74) (%)	Long‐term group (n = 74) (%)	*P*
Operative time ≥8 h	25 (17.5)	13 (17.3)	>0.99	12 (16.2)	13 (17.6)	>0.99
Intraoperative blood loss ≥500 mL	78 (54.5)	30 (40.0)	0.047	37 (50.0)	30 (40.5)	0.32
Postoperative complications (≥C‐D grade II)	59 (41.3)	25 (33.3)	0.31	27 (36.5)	25 (33.8)	0.86
Bile leakage	10 (7.0)	5 (6.7)	>0.99	4 (5.4)	5 (6.8)	>0.99
Infectious complications	27 (18.9)	9 (12.0)	0.25	14 (18.9)	9 (12.2)	0.36
SSI	21 (14.7)	8 (10.7)	0.53	10 (13.5)	8 (10.8)	0.80
Incisional SSI	7 (4.9)	2 (2.7)	0.72	4 (5.4)	2 (2.7)	0.68
Organ/space SSI	18 (12.6)	6 (8.0)	0.37	8 (10.8)	6 (8.1)	0.78
RSI	6 (4.2)	1 (1.3)	0.43	4 (5.4)	1 (1.4)	0.37
Pneumonia	4 (2.8)	0 (0)	0.30	2 (2.7)	0 (0)	0.50
*Clostridioides* colitis	2 (1.4)	0 (0)	0.55	2 (2.7)	0 (0)	0.50
Cholangitis	0 (0)	1 (1.3)	0.34	0 (0)	1 (1.4)	>0.99

Abbreviations: C‐D, Clavien‐Dindo; RSI, remote site infection; SSI, surgical site infection.

Table 5 shows the background characteristics of the patients undergoing LHR in the short‐ and long‐term groups of the unmatched and matched cohorts. In the unmatched cohort, the proportion of major hepatectomy was significantly higher in the long‐term group. After PSM, the study group of 120 patients was well matched. Pathologically, liver cirrhosis was confirmed in 19 patients (31.7%) in the short‐term group and 11 patients (18.3%) in the long‐term group (*P *=* *0.14).

**Table 5:** Background characteristics in patients undergoing laparoscopic hepatic resection

	Unmatched cohort			Matched cohort		
	Short‐term group (n = 122) (%)	Long‐term group (n = 63) (%)	*P*	Short‐term group (n = 60) (%)	Long‐term group (n = 60) (%)	*P*
Age >65 y	84 (68.9)	43 (68.3)	>0.99	36 (60.0)	42 (70.0)	0.34
Gender, male	85 (69.7)	46 (73.0)	0.73	39 (65.0)	44 (73.3)	0.43
Diabetes	46 (37.7)	18 (28.6)	0.26	18 (30.0)	18 (30.0)	>0.99
BMI >25 kg/m^2^	40 (32.8)	22 (34.9)	0.87	20 (33.3)	22 (36.7)	0.85
ASA class ≥3	25 (20.5)	7 (11.1)	0.15	6 (10.0)	7 (11.7)	>0.99
Platelet count <10 × 10^4^/μL	28 (23.0)	13 (20.6)	0.85	14 (23.3)	12 (20.0)	0.83
ALT >30 U/L	57 (46.7)	30 (47.6)	>0.99	38 (63.3)	29 (48.3)	0.14
Child‐Pugh grade B	7 (5.7)	1 (1.6)	0.27	1 (1.7)	1 (1.7)	>0.99
Major hepatectomy	2 (1.6)	5 (7.9)	0.046	2 (3.3)	2 (3.3)	>0.99
Operation type			0.30			0.35
Limited resection	103 (84.4)	51 (81.0)		54 (90.0)	51 (85.0)	
Segmentectomy	3 (2.5)	1 (1.6)		0 (0)	1 (1.7)	
Left lateral sectionectomy	11 (9.0)	6 (9.5)		2 (3.3)	6 (10.0)	
Right anterior sectionectomy	1 (0.8)	0 (0)		1 (1.7)	0 (0)	
Right posterior sectionectomy	2 (1.6)	0 (0)		1 (1.7)	0 (0)	
Hemihepatectomy	2 (1.6)	5 (7.9)		2 (3.3)	2 (3.3)	
Abdominal drainage	108 (88.5)	51 (81.0)	0.24	53 (88.3)	48 (80.0)	0.32

Abbreviations: ALT, alanine aminotransferase; ASA, American Society of Anesthesiologists; BMI, body mass index.

Table 6 shows the perioperative outcomes in patients undergoing LHR in the short‐ and long‐term groups of the unmatched and the matched cohorts. The proportion of longer operative time (≥8 hours) tended to be higher in the long‐term group of the unmatched cohort. However, there were no significant differences in the proportion of patients with longer operative times (≥8 hours; 3.3% vs 10.0%, *P *=* *0.27), greater intraoperative blood loss (≥500 mL; 5.0% vs 8.3%, *P *=* *0.72), or worse postoperative complications (≥Clavien‐Dindo grade II; 8.3% vs 11.7%, *P *=* *0.76) between the short‐ and long‐term groups in the matched cohort. No RSI were reported in either group. The incidence rate of SSI was comparable between the short‐ and long‐term groups (3.3% vs 1.7%, *P *>* *0.99).

**Table 6:** Perioperative outcomes in patients undergoing laparoscopic hepatic resection

	Unmatched cohort			Matched cohort		
	Short‐term group (n = 122) (%)	Long‐term group (n = 63) (%)	*P*	Short‐term group (n = 60) (%)	Long‐term group (n = 60) (%)	*P*
Operative time ≥8 h	3 (2.5)	6 (9.5)	0.064	2 (3.3)	6 (10.0)	0.27
Intraoperative blood loss ≥500 mL	4 (3.3)	5 (7.9)	0.28	3 (5.0)	5 (8.3)	0.72
Postoperative complications (≥C‐D grade II)	9 (7.4)	7 (11.1)	0.42	5 (8.3)	7 (11.7)	0.76
Bile leakage	3 (2.5)	1 (1.6)	>0.99	3 (5.0)	1 (1.7)	0.62
Infectious complications	2 (1.6)	1 (1.6)	>0.99	2 (3.3)	1 (1.7)	>0.99
SSI	2 (1.6)	1 (1.6)	>0.99	2 (3.3)	1 (1.7)	>0.99
Incisional SSI	0 (0)	0 (0)	NA	0 (0)	0 (0)	NA
Organ/space SSI	2 (1.6)	1 (1.6)	>0.99	2 (3.3)	1 (1.7)	>0.99
RSI	0 (0)	0 (0)	NA	0 (0)	0 (0)	NA

Abbreviations: C‐D, Clavien‐Dindo; NA, not assessed; RSI, remote site infection; SSI, surgical site infection.

## 4 DISCUSSION

The current study evaluated the efficacy of giving short‐term prophylactic antibiotics (before and until 24 hours after surgery) that were discontinued within 24 hours after surgery in patients undergoing OHR and in those undergoing LHR. No significant differences in the incident rates of infectious complications were found between the short‐ and long‐term groups, and the rate of SSI was not significantly different between the OHR and LHR groups. Giving short‐term prophylactic antibiotics was not associated with an increase in PIC in patients undergoing LHR.

Prophylactic antibiotics aim at reducing the microbial burden of intraoperative contamination. To maximize the prophylactic effect of antimicrobial agents, maintaining therapeutic levels of the agent in both serum and tissues throughout the operation is necessary.^8^ However, long‐term antimicrobial prophylaxis contributes to increased drug resistance and risk of *C. difficile* disease.^10^ Giving short‐term prophylactic antibiotics until 24 hours after surgery was recommended in several guidelines.^9^, ^10^, ^11^ In Japan, following the findings of a nationwide randomized controlled study,^15^ the guidelines provided by JSSI and JSC recommend giving prophylactic antibiotics 1 hour before surgery, with redosing every 3 hours during surgery and dosing until 24 hours after surgery in patients undergoing hepatic resection without biliary tract reconstruction.^16^ Several studies also showed that short‐term antimicrobial prophylaxis was sufficient for hepatic resection. Togo et al^5^ compared patients treated with antimicrobial agents for 2 days until POD 1 to those treated for 5 days until POD 4 and showed that giving prophylactic antibiotics for 2 days was sufficient. Sakoda et al^27^ compared postoperative antimicrobial treatment given only once after surgery with that continued until 3 days after surgery and found that giving antibiotics for 3 days had no additional benefit. In the current study, the efficacy of giving short‐term antibiotics, initiated before surgery and continued until 24 hours after surgery, was confirmed in patients with OHR. Conversely, Zhou et al^7^ reported no significant difference in the SSI rates between patients treated with prophylactic antibiotics before skin incision only and those who were untreated. However, the SSI incidence rate of 14.2% in that study was relatively higher than that in the current study (7.9%). Therefore, giving prophylactic antibiotics might be necessary to prevent SSI development after hepatic resection.

The cohort of the nationwide randomized controlled study evaluating the efficacy of giving short‐term antibiotics in Japan was limited to patients undergoing OHR, and the validity of giving short‐term prophylactic antibiotics in those undergoing LHR has not been evaluated, although LHR is increasingly adopted worldwide. The advantages of LHR over OHR are lower blood loss and morbidities including SSI, which were reportedly explained by meticulous liver dissection under better magnification, reduced intractable ascites by small mobilization of the liver, and small surgical wound.^17^, ^18^, ^19^ In the current study, LHR was used in 185 patients (45.9%), and the rates of intraoperative blood loss ≥500 mL and postoperative complications including SSI were lower compared with those in patients undergoing OHR. Several guidelines also indicate age, obesity, and glucose control as the risk factors for SSI.^10^, ^12^, ^16^ Among the risk factors for hepatic resection are high ASA score, liver cirrhosis, prolonged operation, repeat hepatectomy, and bile leakage.^17^, ^28^ In the current study, the proportion of major hepatectomy was significantly higher in the long‐term group among patients undergoing LHR before PSM. However, following the PSM analysis, no imbalance was identified in the background characteristics between the short‐ and long‐term groups, and giving short‐term prophylactic antibiotics was not associated with an increase in PIC in patients undergoing LHR.

In a randomized controlled trial, Hirokawa et al^6^ showed the efficacy of prophylactic antibiotics during operation alone compared with prophylactic antibiotics for 3 days after hepatic resection. In the current study, the SSI incidence rate was 1.6% in the short‐term group receiving prophylactic antibiotics before and until 24 hours after surgery among those undergoing LHR, which tended to be lower than that in patients undergoing OHR (14.7%) in the unmatched cohort. Therefore, giving prophylactic antibiotics before and during operation alone can be sufficient in patients undergoing LHR. A nationwide randomized controlled study is necessary to confirm the efficacy of prophylactic antibiotics before and during operation alone for patients undergoing LHR.

*Clostridioides* colitis developed in one patient in the short‐term group. Giving antimicrobials could be a risk factor for *C. difficile* disease. However, there was no significant difference in the incidence rate of *Clostridioides* colitis between the short‐ and long‐term groups, and *Clostridioides* colitis was not observed in the long‐term group. Risk factors for *C. difficile‐*associated diarrhea include renal failure, hospital admission, and use of proton pump inhibitors.^29^ The two patients were given proton pump inhibitors starting from POD 2 and developed diarrhea at POD 7. Therefore, the use of proton pump inhibitors might contribute to the development of *Clostridioides* colitis.

Guidelines by the JSSI and the JSC recommend cefazolin, cefotiam, FMOX, and sulbactam/ampicillin as prophylactic antibiotics in hepatic resection without biliary tract reconstruction. FMOX is a second‐generation cephem antibiotic with antibacterial activity against airborne bacteria including *Staphylococcus aureus*, enteric bacteria, *Escherichia coli*, and *Klebsiella pneumoniae* and anaerobic bacteria including *Bacteroides fragilis*. The antibacterial activity of FMOX is superior to cefazolin and cefmetazole with low toxicity.^30^ In the current study, *S. aureus*,* Enterococcus* species, and *Enterobacter cloacae* were detected in patients with incisional SSI. Therefore, FMOX was considered an appropriate prophylactic antimicrobial agent.

Methicillin‐resistant *Staphylococcus aureus* was detected in four patients (1.0%) who developed incisional SSI with or without organ/space SSI. In previous reports, the incident rate of MRSA‐related infections after hepatic resection was approximately 2.4%‐4%.^6,^ 27 The incidence rate of MRSA‐related SSI in the current study was rather lower than those reported previously. The strict compliance with standard CDC precautions by both the physicians and nurses at the study hospital might have contributed to the low incidence of MRSA‐related SSI in the study cohort.

As a limitation, the retrospective nature of the current study might have resulted in a certain level of bias in patient enrolment even after careful PSM analysis. However, the study included a large number of patients with liver tumors including malignant and benign diseases. The significance of short‐term antimicrobial prophylaxis might be properly evaluated in patients undergoing hepatic resection.

In conclusion, giving prophylactic antibiotics before surgery, at 3‐h intervals during surgery, and until 24 hours after surgery for hepatic resection, as indicated by the guidelines of JSSI and JSC, was sufficient in preventing PIC in patients undergoing OHR as well as in those undergoing LHR.

## DISCLOSURE

Funding: This work was supported by the Health, Labour and Welfare Policy Research Grants from the Ministry of Health, Labour, and Welfare of Japan (Policy Research for Hepatitis Measures [H30‐Kansei‐Shitei‐003]).

Conflicts of Interest: Authors declare no conflicts of interest for this article.

Author Contribution: Hiroji Shinkawa, Shigekazu Takemura, Ryosuke Amano, Kenjiro Kimura, Takayoshi Nishioka, Tokuji Ito, Toru Miyazaki, and Atsushi Ishihara have 1) substantial contributions to the conception and analysis of data for work, and 2) drafted the work.Shogo Tanaka and Shoji Kubo have 1) substantial contributions to the conception and analysis of data for work, and 2) critically revised the draft for important intellectual content.
